# Inhibitory effects of *N‐trans*‐cinnamoyltyramine on growth of invasive weeds and weedy rice

**DOI:** 10.1002/pei3.70017

**Published:** 2024-11-03

**Authors:** Vang V. Le, Ay V. Nguyen, Danh T. Luu, Felix B. Fritschi, Cuong T. Nguyen, Thi L. Ho

**Affiliations:** ^1^ College of Agriculture Can Tho University Can Tho Vietnam; ^2^ Division of Plant Science and Technology University of Missouri Columbia Missouri USA; ^3^ Cuu Long Delta Rice Research Institute Can Tho Vietnam

**Keywords:** allelopathic rice, dose‐dependent response, eco‐friendly herbicides, *N‐trans*‐cinnamoyltyramine, plant growth inhibition

## Abstract

*N‐trans*‐cinnamoyltyramine (NTCT) has been identified from an allelopathic Vietnamese rice accession OM 5930. This study employed bioassays to analyze NTCT's effects on shoot and root growth of multiple test and weed species. NTCT demonstrated potent inhibitory effects on cress, lettuce, canola, palmer amaranth, timothy, barnyardgrass, red sprangletop, and weedy rice, with increasing concentrations leading to substantial reductions in growth in all species. Linear regression analysis of dose response curves revealed ED_50_ values for NTCT, providing critical insights into the concentration required for 50% growth inhibition in each species. They revealed high sensitivity of the test species cress and lettuce, intermediate sensitivities of barnyardgrass, red sprangletop, timothy, and amaranth, and comparatively lower sensitivity of two weedy rice accessions. The findings underscore NTCT's efficacy in suppressing the growth of a wide range of weeds, including both grasses and broadleaf species. As such, NTCT may hold promise as a tool for sustainable weed management, particularly in addressing herbicide‐resistant weeds in diverse ecological settings.

## Graphical abstract

This figure illustrates the inhibitory effects of NTCT, a compound found in an allelopathic Vietname rice cultivar OM 5930, on various weed species and crops. NTCT, produced by rice roots, demonstrates strong allelopathic activity, targeting invasive weeds such as barnyardgrass (*Echinochloa crus‐galli*), weedy rice (*Oryza sativa* f. *spontanea*), Palmer amaranth (*Amaranthus palmeri*), and others, by interfering with their growth. This mechanism highlights NTCT's potential as a bio‐herbicide for sustainable weed management in rice cultivation.

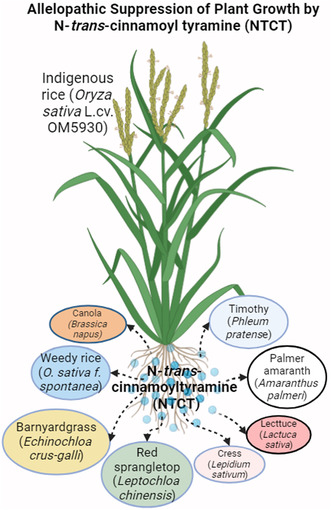

## 1 INTRODUCTION

Weed species present formidable threats to global crop production, causing economic losses and jeopardizing food security across diverse climates (Ramesh et al., 2017). This study focused on weedy rice (*Oryza sativa* cv. *spontanea*) and four invasive weeds noted for their aggressive nature and detrimental impacts on crops: Palmer amaranth (*Amaranthus palmeri*), timothy (*Phleum pratense*), barnyardgrass (*Echinochloa crus‐galli*), and red sprangletop (*Leptochloa chinensis*). Palmer amaranth, originating from the southwestern United States, poses a major threat to various crops, including cotton (*Gossypium hirsutum*), soybeans (*Glycine max*), and maize (*Zea mays*), and is rapidly spreading to other regions (Culpepper et al., 2006). Timothy, a European grass, invades agricultural ecosystems, diminishing yields and forage quality, especially in pasture and grassland areas (DiTomaso, 2000). Barnyardgrass, particularly troublesome in rice fields, competes intensely with rice plants for nutrients and develops resistance to herbicides, necessitating integrated weed management (Kacan et al., 2020; Pinsupa et al., 2022). Red sprangletop infests Asian rice fields, presenting challenges for effective control and negatively impacting crop yield and quality (Chin, 2001; Wang et al., 2022). Weedy rice has emerged as a substantial threat to rice production in Arkansas and the broader United States. It mimicks cultivated rice and poses a distinctive challenge by competing for resources, resulting in yield losses and reduced crop quality (Delouche et al., 2007; Jia & Gealy, 2018).

Canola (*Brassica napus*), cress (*Lepidium sativum*), and lettuce (*Lactuca sativa*) are commonly used in allelopathy studies due to their sensitivity to allelopathic substances (Duke et al., 2005; Zubair et al., 2017). Canola, known for its prolific seed production, is often chosen to assess the effects of allelochemicals on crop species (Duke et al., 2005). Cress, a fast‐growing plant, provides rapid insights into physiological responses to allelopathic compounds (Alsaadawi et al., 2012; Tran et al., 2016). Lettuce, frequently used in bioassays, is particularly effective for early detection of allelopathic interactions (Farooq et al., 2013; Kong et al., 2019).

Over the past few decades, the exploration of allelochemicals as a natural alternative to synthetic herbicides has gained increasing momentum in agricultural research. This shift is largely driven by the urgent need to address the growing problem of herbicide‐resistant weeds, a challenge that has escalated globally (Duke et al., 2000; Ofosu et al., 2023). Unlike conventional herbicides, which typically have a single mode of action, allelochemicals operate by targeting multiple biochemical pathways. This feature makes them particularly promising in preventing the evolution of resistance (Einhellig, 1996; Weston & Duke, 2003). Such multi‐site activity provides a sustainable strategy for weed management, particularly in rice cultivation, where herbicide resistance in key weed species like *Echinochloa crus‐galli* and *Leptochloa chinensis* poses a significant threat (Chauhan & Johnson, 2011; Zhang et al., 2022). Research into the allelopathic potential of crops such as sorghum (*Sorghum bicolor*) and sunflower (*Helianthus annuus*) has demonstrated their ability to reduce weed biomass in major cereals, including rice (*Oryza sativa*) and wheat (*Triticum aestivum*) (Belz, 2007; Das et al., 2021; Soltys et al., 2013). More recently, focus has increasingly shifted to the allelopathic properties inherent in rice itself (Rahaman et al., 2022). Earlier study revealed that rice varieties could exhibit strong allelopathic effects, suppressing the growth of problematic weed species through root exudates and residue decomposition (Olofsdotter et al. 2008). This discovery has opened new avenues for breeding rice varieties with enhanced allelopathic potential, presenting an integrated approach to weed management (Eroğlu et al., 2021; Abbas et al., 2021).

Globally, rice allelopathy research has expanded, particularly in regions where continuous rice cropping has intensified weed pressure and heightened dependence on chemical herbicides. A growing body of research has identified various allelopathic compounds in rice, such as momilactones and phenolic acids, which effectively inhibit weed germination and growth (Khanh et al., 2007; Scavo & Mauromicale, 2021). For example, rice varieties from Southeast Asia have demonstrated significant allelopathic effects in suppressing barnyard grass (*Echinochloa crus‐galli*), a notorious weed in paddy systems (Xuan et al., 2005; Abbas et al., 2021). These discoveries have sparked global efforts to incorporate such traits into new rice cultivars, aligning with initiatives aimed at reducing the use of synthetic herbicides and promoting more sustainable agricultural practices (Jabran et al., 2015; Macías et al., 2019). Looking ahead, rice allelopathy holds dual benefits: enhancing weed suppression while contributing to the global shift towards sustainable agriculture. By leveraging the natural allelopathic properties of rice, farmers can reduce their reliance on synthetic herbicides, lower production costs, and minimize environmental risks. Furthermore, the integration of allelopathic rice varieties into cropping systems offers a promising solution to the global rise in herbicide‐resistant weeds, making it a critical component of future weed management strategies (Aci et al., 2022; Duke et al., 2022).

Rice allelopathy, involving the synthesis and release of bio‐based allelochemicals like alkaloids, phenolics, flavonoids, terpenes, and glucosinolates, earlier emerged as an eco‐friendly option for weed control in paddy ecosystems (Kim & Shin, 2008). Previous studies have explored allelochemicals from different parts of the rice plant, including leaves, stems, and roots, using organic solvents (Blum, 1998; Chou et al., 1991; Inderjit, 1996). The *β*‐phenylethylamine, *N‐trans*‐cinnamoyltyramine (NTCT), identified as a promising allelochemical in rice (Ho et al., 2021; Le Thi et al., 2014; Thi et al., 2014, 2017), remains insufficiently investigated for its efficacy on diverse invasive weeds from tropical and temperate regions. This study aims to bridge this gap by investigating NTCT inhibitory activity on mono‐ and di‐cotyledonous species, including canola, cress, lettuce, palmer amaranth, timothy, weedy rice, barnyardgrass, and red sprangletop.

## 2 MATERIALS AND METHODS

Seeds of PI 653426 and PI 653431, two specific weedy rice accessions, were acquired from the Dale Bumpers National Rice Research Center, Stuttgart, Arkansas, USA. Seeds were securely stored in the Crop Physiology Lab of the Division of Plant Sciences at the University of Missouri, Missouri, USA, and then in the Biotechnology in Plant Protection lab, 5.20 ATL, Can Tho University, Can Tho, Vietnam, and were used for experiments as needed. Palmer amaranth, timothy, canola, cress, and lettuce seeds were obtained from Johnny's Selected Seeds, Waterville, ME 04903, USA. Seeds of barnyardgrass and red sprangletop were collected at maturity from experimental fields at the Cuu Long Delta Rice Research Institute (CLRRI) and dried in an incubator (Forced Convection Laboratory Incubators, Esco Isotherm) at 50°C for 16 h, maintained at room temperature (25 ± 1°C) for 1 h, followed by storage at 4°C until used.

### 2.1 Activity and allelopathy of synthesized NTCT from rice

NTCT (5 mg) was synthesized in the Division of Plant Sciences at the University of Missouri, USA, and dissolved in 5 mL of methanol (Thi et al., 2017). NTCT concentrations of 0.024, 0.24, 2.4, 4.8, 9.6, and 24 μM were added onto filter paper sheets in Petri dishes (3 cm), and then the Petri dishes were placed in a fume hood (Kewaunee Scientific Corporation, 2700 West Front Street Statesville, NC 28677, USA) at room temperature until the methanol evaporated (about 1–1.5 h). Following methanol evaporation, the dry filter papers with NTCT substance were moistened with distilled H_2_O (1.0 mL for the Petri dish). Ten pre‐germinated seeds of a target species, such as palmer amaranth, timothy, PI 653426, and PI 653431 weedy rice, barnyardgrass, red sprangletop, canola, cress, and lettuce were then placed onto the moistened filter paper in each Petri dish. Controls consisted of filter paper moistened with distilled H_2_O (1.0 mL) without NTCT. Pre‐germinated seeds for the bioassay were generated by soaking them in distilled water for 24–48 hrs, and then incubating them at 32–35°C for an additional 24–72 h. The pre‐germinated seeds were transferred to Petri dishes with or without NTCT and were incubated at 25°C for 48 h in the dark. At the end of this incubation, root and shoot lengths of all plants were measured. The bioassays were conducted twice using a completely randomized design with three replications following the bioassay procedure described by Thi et al. (2014).

### 2.2 Data analysis

For the bioassays using the different concentrations of NTCT, root or shoot length data from each well of 24‐well plates or Petri dish were averaged. The Dose–Response Curves (DRCs) package within the R statistical software was used to fit the root and shoot length data (Ritz et al., 2015). To determine the best fit model from a range of options (e.g., linear regression, Weibull, Log‐logistic, Cubic, Quadratic), the “MSELECT” function was utilized. The identified best fit model was the four‐parameter log‐logistic function LL4, represented as $y=c+\frac{d-c}{1+\exp\left(b\left(\log\left(x\right)-\log\left(e\right)\right)\right)}$. Subsequently, this model was employed to ascertain the 10%, 50%, and 90% inhibitory concentrations (IC_10_, IC_50_ and IC_90_) using the effective dose (ED) function integrated in the DRC package (the ED_10_, ED_50_, and ED_90_). In the equation, y denotes the inhibition (%), *x* represents concentration (μM), and parameters *b*, *c*, *d*, and *e* are assumed to be independently distributed from normal distributions with constant variance. The two nested models, e.g., simple with less parameters and complex models with more parameters, were compared using *F*‐tests to determine which model provided the better fit for the data.

## 3 RESULTS

### 3.1 Inhibitory activity of NTCT on indicator test plant species

**FIGURE 1:**
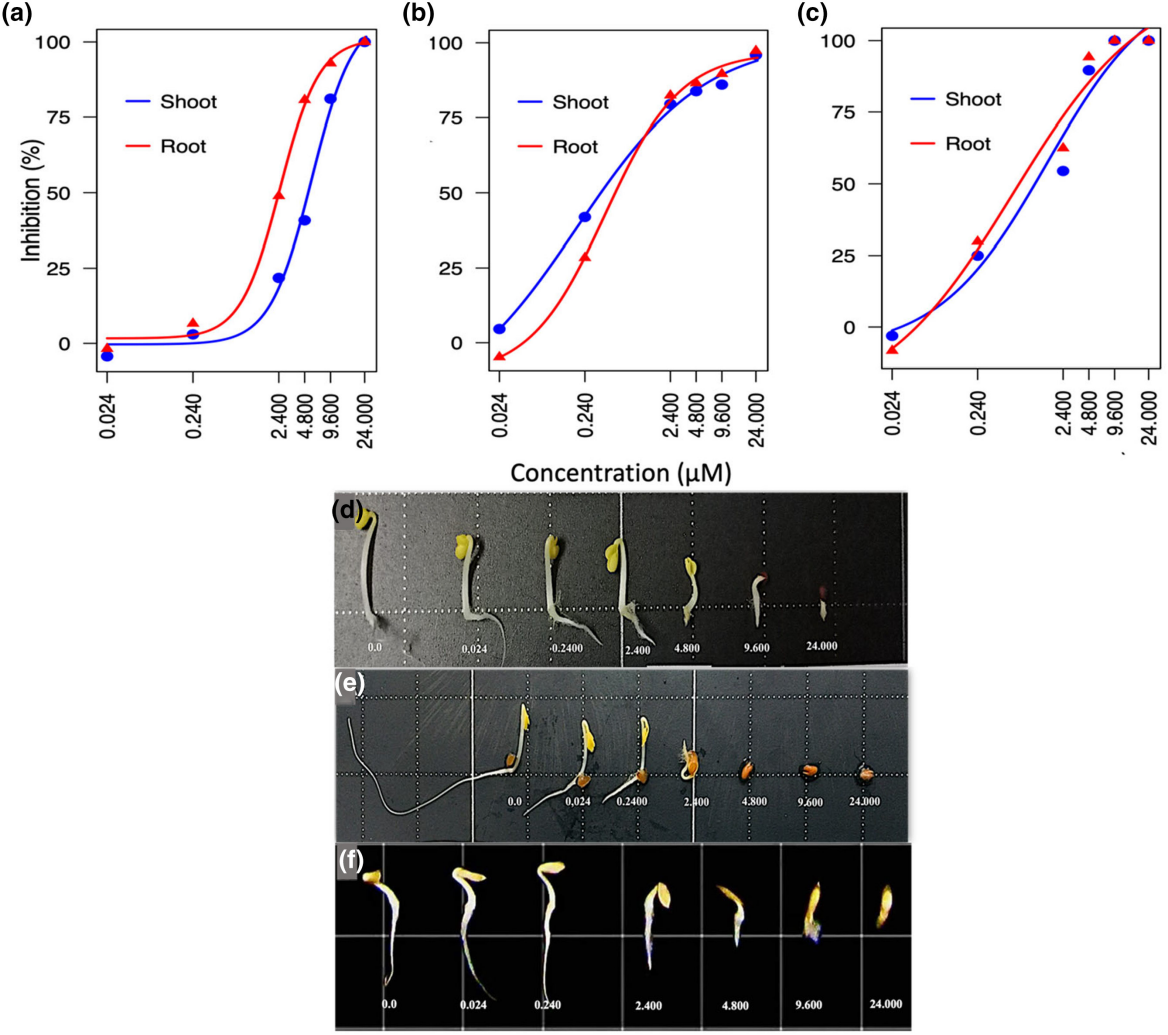
Effect of *N‐trans*‐cinnamoyltyramine (NTCT) on the shoot and root growth of Canola (*Brassica napus*) (a, d), Cress (*Lepidium sativum*) (b, e) and Lettuce (*Lactuca sativa*) (c, f) at concentrations of 0.024, 0.24, 2.4, 4.8, 9.6 and 24 μM. Dose–response curves based on analysis using the “drc” package in R.

The allelopathic effects of NTCT on the shoot and root growth of canola, cress, and lettuce were investigated across a range of concentrations from 0.024 to 24 μM. The average percentage change in shoot and root growth at each concentration are presented in Figure 1. The data reveals a progressive decline in both shoot and root growth of canola plants in response to increasing concentrations of NTCT. At the second lowest concentration of 0.24 μM, canola exhibited a slight decrease in shoot and root growth (3.0% and 6.6%) compared to the control, indicating a mild inhibitory effect. However, as the concentration of NTCT increased, the inhibitory effects on both shoot and root growth became increasingly severe. At 9.6 μM NTCT shoot growth was inhibited by 81.1% and root growth by 92.9%. Furthermore, at 24 μM, both shoot and root growth were completely inhibited (Figure 1a,d).

Similar to canola, cress plants displayed a dose‐dependent response to NTCT treatment with no (root) or minimal (shoot) effect on growth at 0.024 μM, a moderate inhibitory effect at 0.24 μM (41.9% shoot and 28.4% root). With a change in NTCT concentration from 0.24 to 2.4 μM, growth inhibition more than doubled for roots and nearly doubled for shoots. Additional increments of growth inhibition associated with NTCT concentrations beyond 2.4 μM were relatively small, but, at 24 μM NTCT, root and shoot growth inhibition averaged 94.8%, indicating nearly complete inhibition (Figure 1b,e).

Similar to canola, no significant inhibition of root or shoot growth was found in lettuce at the lowest NTCT concentration. However, unlike canola which showed a limited growth inhibition at 0.24 μM, both root and shoot growth of lettuce were inhibited by approximately 25% at that same concentration. With further increases in NTCT, root and shoot growth inhibition became more severe, reaching 89.6% and 94.2% inhibition at 4.8 μM, respectively, and complete suppression of growth at 9.6 and 24 μM. These results demonstrate the potent inhibitory effects of NTCT on root and shoot growth of canola, cress, and lettuce plants, with a generally greater sensitivity of root than shoot growth in canola but more similar effects on growth of root and shoot in lettuce (Figure 1e,f).

### 3.2 Inhibitory activity of NTCT on temperate weed species

**FIGURE 2:**
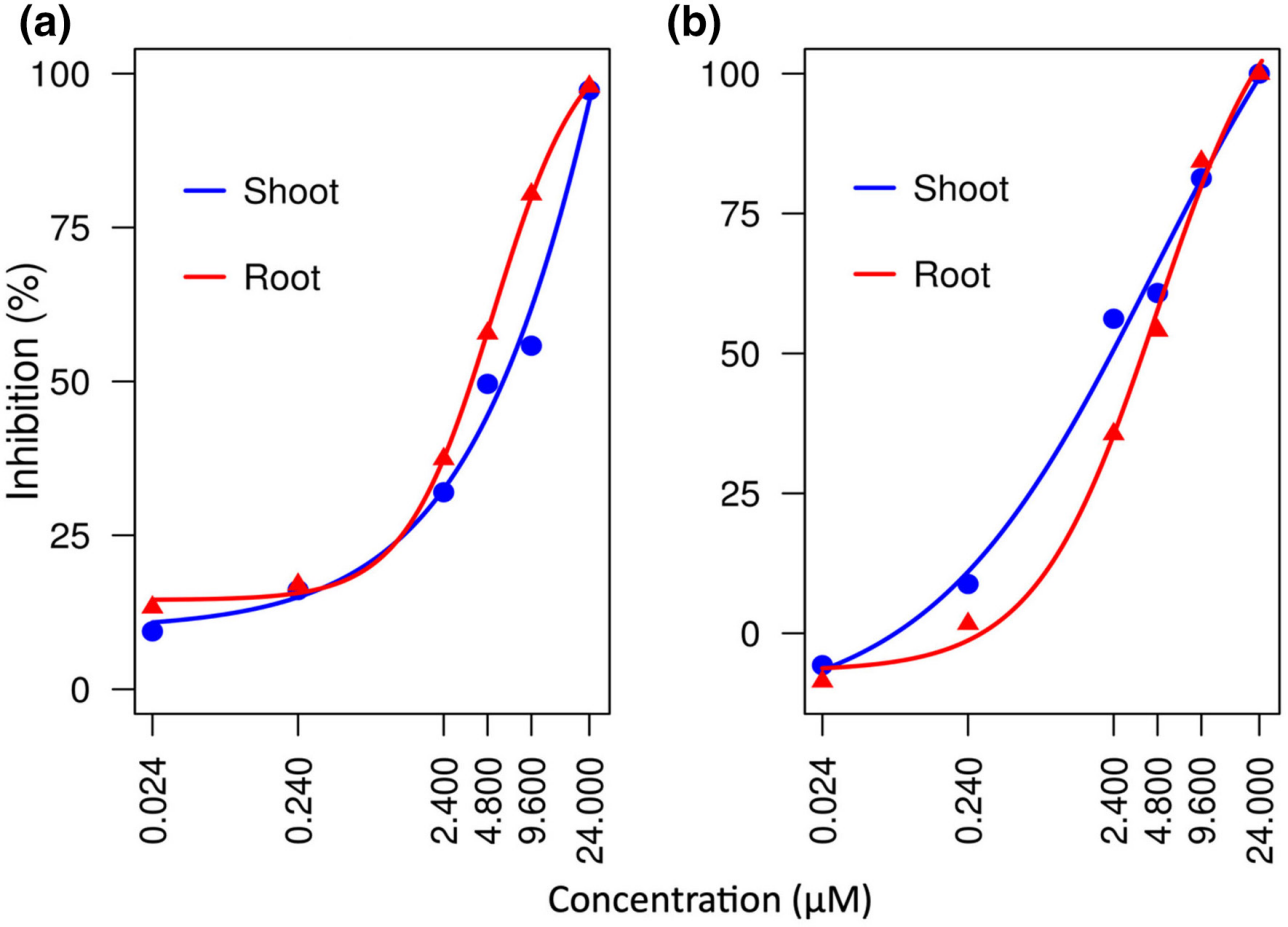
Effect of *N‐trans*‐cinnamoyltyramine (NTCT) on the shoot and root growth of palmer amaranth (*Amaranthus palmeri*) (A) timothy (*Phleum pratense*) (B) at concentrations of 0.024, 0.24, 2.4, 4.8, 9.6 and 24 μM. Dose–response curves based on analysis using the “drc” package in R.

The impact of NTCT on shoot and root growth of palmer amaranth and timothy was assessed across a spectrum of NTCT concentrations. Shoot and root growth of palmer amaranth were sensitive to NTCT at very low concentrations, with 0.024 μM limiting shoot and root growth to 90.6% and 86.7% of the control, respectively. However, a substantial increase in NTCT concentration to 4.8 μM was needed to inhibit palmer amaranth root and shoot growth by more than 50%. Nonetheless, at the highest concentration of 24 μM, shoot growth plummeted to a mere 2.7% and root growth to 2.1% of the control, equivalent to 97.3% and 97.9% of inhibition (Figure 2a).

In contrast to palmer amaranth, timothy growth was not inhibited by 0.024 μM NTCT. Interestingly, shoot growth (8.8% inhibition) was more sensitive to low concentrations of NTCT than root growth (1.7% inhibition) which was relatively unaffected at 0.24 μM. However, as the NTCT concentration increased beyond 2.4 μM, both shoot and root growth declined sharply, reaching 100% of inhibition at 24 μM (Figure 2b).

### 3.3 Inhibitory activity of NTCT on weedy rice

**FIGURE 3:**
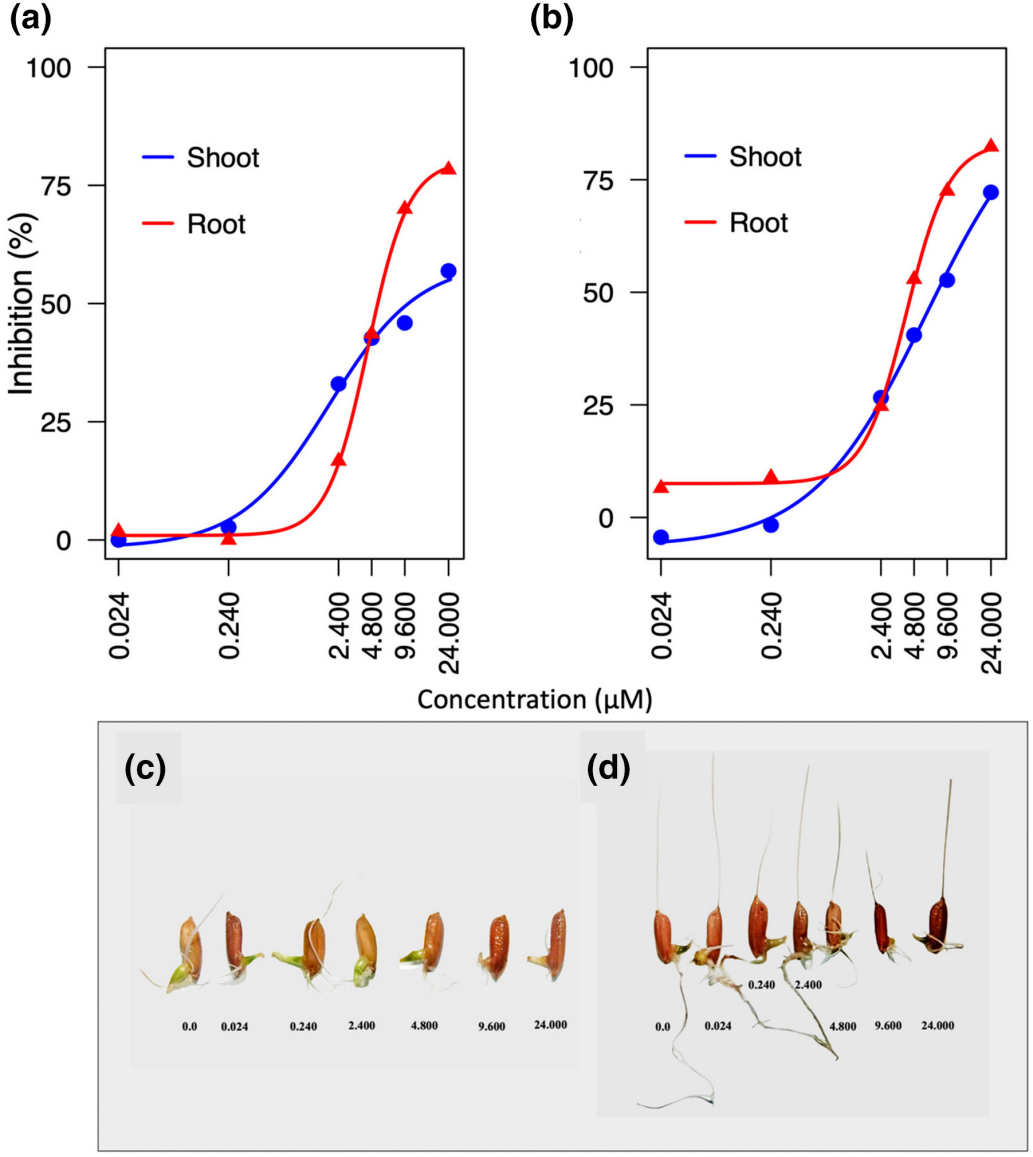
Effect of *N‐trans*‐cinnamoyltyramine (NTCT) on the shoot and root growth of weedy rice PI 653426 (a, c) and PI 653431 (b, d) at concentrations of 0.024, 0.24, 2.4, 4.8, 9.6 and 24 μM. Dose–response curves based on analysis using the “drc” package in R.

By examining the response of weedy rice accessions to varying concentrations of NTCT, valuable insights into its potential as a growth inhibitor and its suitability for weed management in rice cultivation may be gained. To this end, the effects of NTCT on shoot and root growth of two distinct weedy rice accessions, namely PI 653426 and PI 653431, were examined. Both PIs exhibited notable dose‐dependent effects of NTCT on both shoot and root growth (Figure 3). In the case of PI 653426, neither shoot nor root growth was influenced much by NTCT concentrations of 0.24 μM or lower. Increasing concentrations beyond that level caused increasing levels of shoot and root growth inhibition, reaching a maximum inhibition of 57.6% of shoot and 77.9% of root growth at the highest NTCT concentration tested (24 μM). Although root growth remained relatively unaffected at lower concentrations (0.024 and 0.24 μM), a pronounced decline was observed at higher concentrations and was associated with a greater sensitivity of root growth than that of shoot growth (Figure 3a,c).

The response of PI 653431 to NTCT differed somewhat from that of PI 653426 in that root but not shoot growth was slightly inhibited at low concentrations (0.024 and 0.24 μM). Greater sensitivity of root growth than shoot growth largely persisted with increasing NTCT concentrations. At the highest concentration (24 μM), root growth was inhibited by 82.3% and shoot growth by 72.2% Overall, the range of NTCT concentrations caused similar significant responses in both weedy rice lines, with root growth exhibiting a greater sensitivity than shoot growth at high NTCT concentrations (Figure 3b,d).

### 3.4 Inhibitory activity of NTCT on tropical and sub‐tropical weed species

**FIGURE 4:**
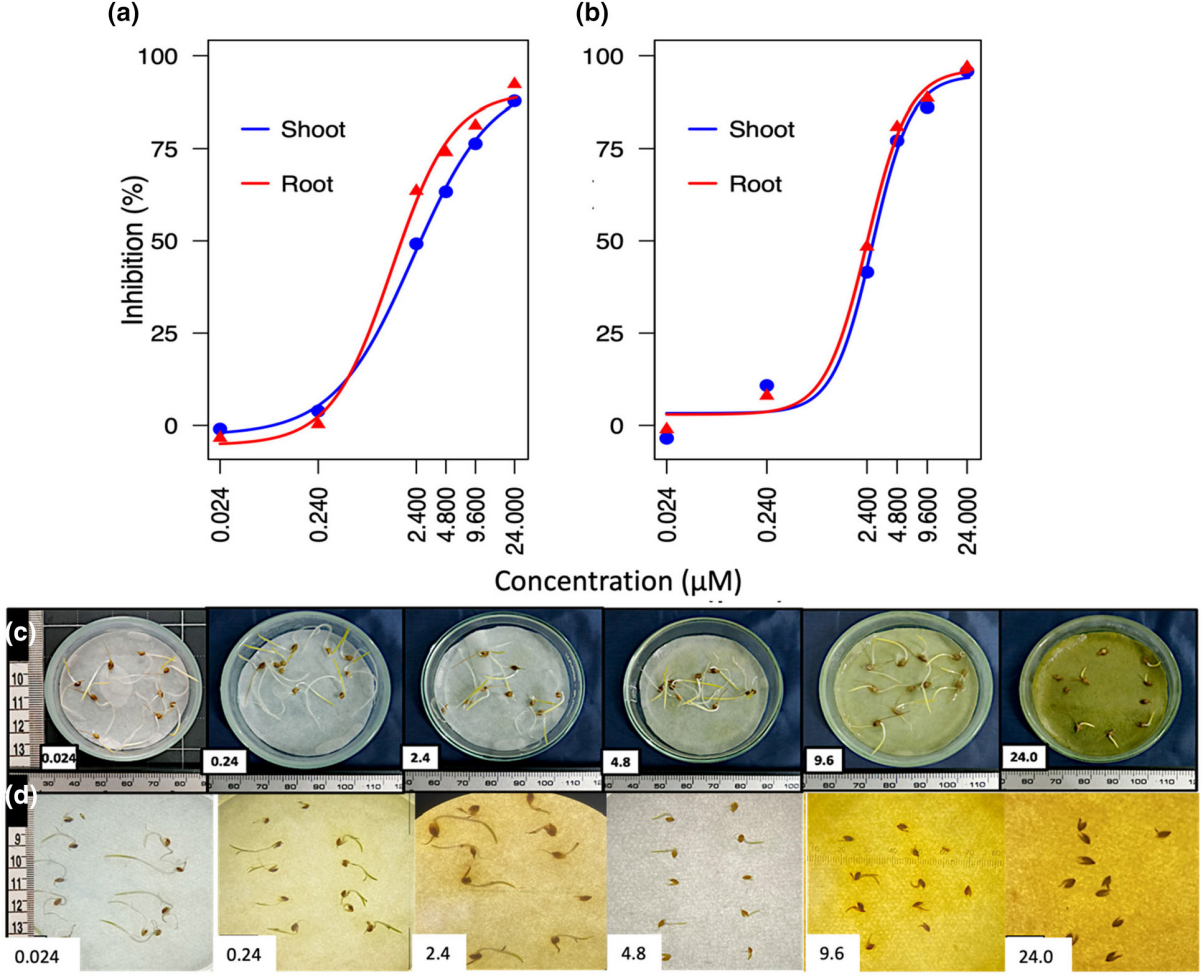
Effect of *N‐trans*‐cinnamoyltyramine (NTCT) on shoot and root growth of barnyardgrass (*Echinochloa crus‐galli*) (a, c) and red sprangletop (*Leptochloa chinensis*) (b, d) at concentrations of 0.024, 0.24, 2.4, 4.8, 9.6 and 24 μM. Dose–response curves based on analysis using the “drc” package in R.

The impact of NTCT on barnyardgrass and red sprangletop shoot and root growth was examined across concentrations ranging from 0.024 to 24 μM (Figure 4). Shoot and root growth were strongly influenced by increasing NTCT concentrations in both species. Barnyardgrass shoot and root growth were not sensitive to low NTCT concentrations of 0.024 and 0.24 μM, but, at 2.4 μM, root and shoot growth were inhibited by 48.4% and 41.5%, respectively. Greater NTCT concentration increasingly limited growth, which, at 24 μM NTCT, was only 3.1% and 4.2% that of roots and shoots in the control, equivalent to 96.9% and 95.8% of inhibition, respectively (Figure 4a,c).

The relative impact of NTCT on red sprangletop root and shoot growth was nearly identical at every concentration. While growth was unaffected at 0.024 μM, strong growth reductions were caused by concentrations of 2.4 μM (average inhibition of shoot and root growth was 56.9%) and higher. At 24 μM, shoot growth was inhibited by 87.8% and root growth by 92.3% (Figure 4b,d).

### 3.5 Effective dose values of NTCT for root and shoot growth inhibition of six plant species

The effective dose (ED) values obtained from the best‐fit equations using the LL.4 model provide insights into the concentration of NTCT required to achieve specified levels of inhibition in shoot and root growth across eight plant species (Table 1). Canola (*Brassica napus*) showed moderate sensitivity to NTCT. The ED_50_ values were 5.29 μM for shoots and 2.40 μM for roots, indicating that canola shoots require higher concentrations for inhibition compared to roots. The ED_10_ values were 1.63 μM for shoots and 0.70 μM for roots, with the ED_90_ values being 13.7 μM for shoots and 7.39 μM for roots. This indicates that canola roots are more susceptible to NTCT, with inhibition occurring at lower doses compared to shoots. The high ED_90_ values for both tissues imply that near‐complete inhibition requires high NTCT concentrations.

**TABLE 1:** Effective dose (ED) of *N‐trans*‐cinnamoyltyramine (NTCT) on the mean shoot and root growth of eight test plant species.

Species	Tissue	Equation (LL.4)	*R*‐square	ED_10_ (SE)^a^	ED_50_ (SE)	ED_90_ (SE)
				μM		
Canola	Shoot	*y* = −0.34+$\frac{109.2+0.34}{1+\exp\left(-1.8\left(\log\left(x\right)-\log\left(5.8\right)\right)\right)}$	0.99	1.63 (±0.49)	5.29 (±0.84)	13.7 (±5.0)
	Root	*y* = 1.67+$\frac{101.1-1.67}{1+\exp\left(-1.9\left(\log\left(x\right)-\log\left(2.4\right)\right)\right)}$	0.99	0.70 (±0.29)	2.40 (±0.23)	7.39 (±2.41)
Cress	Shoot	*y* = −24.2+$\frac{101+24.2}{1+\exp\left(-0.58\left(\log\left(x\right)-\log\left(0.19\right)\right)\right)}$	0.99	0.036 (±0.045)	0.37 (±0.19)	10.8 (±6.8)
	Root	*y* = −11+$\frac{96.9+11}{1+\exp\left(-0.98\left(\log\left(x\right)-\log\left(0.41\right)\right)\right)}$	0.99	0.098 (±0.03)	0.54 (±0.09)	6.38 (±3.3)
Lettuce	Shoot	*y* = −8.3+$\frac{122.7+8.3}{1+\exp\left(-0.68\left(\log\left(x\right)-\log\left(1.55\right)\right)\right)}$	0.97	0.11 (±0.26)	1.12 (±1.15)	7.75 (±3.08)
	Root	*y* = −28.8+$\frac{122.3+28.8}{1+\exp\left(-0.55\left(\log\left(x\right)-\log\left(0.63\right)\right)\right)}$	0.97	0.092 (±0.21)	0.73 (±0.87)	6.56 (±2.51)
Amaranth	Shoot	*y* = 9.7+$\frac{401.6-9.7}{1+\exp\left(-0.66\left(\log\left(x\right)-\log\left(166.1\right)\right)\right)}$	0.98	0.003 (±0.005)	6.15 (±3.6)	21.10 (±5.23)
	Root	*y* = 14.5+$\frac{107.6-14.5}{1+\exp\left(-1.43\left(\log\left(x\right)-\log\left(5.25\right)\right)\right)}$	0.99	–	3.73 (±0.2)	14.5 (±2.2)
Timothy	Shoot	*y* = −14.5+$\frac{144\mp 14.5}{1+\exp\left(-0.56\left(\log\left(x\right)-\log\left(4.6\right)\right)\right)}$	0.99	0.22 (±0.17)	2.33 (±2.11)	14.7 (±4.9)
	Root	*y* = −6.7+$\frac{121.7+6.7}{1+\exp\left(-1.04\left(\log\left(x\right)-\log\left(4.76\right)\right)\right)}$	0.99	0.77 (±0.3)	3.81 (±1.08)	13.9 (±5.4)
Weedy rice PI 653426	Shoot	*y* = −1.6+$\frac{58.9+1.6}{1+\exp\left(-1.06\left(\log\left(x\right)-\log\left(1.98\right)\right)\right)}$	0.99	0.5 (±0.16)	10.33 (±6.7)	–
	Root	*y* = 0.96+$\frac{80.2-0.96}{1+\exp\left(-2.35\left(\log\left(x\right)-\log\left(4.4\right)\right)\right)}$	0.99	1.85 (±0.11)	5.44 (±0.22)	–
Weedy rice PI 653431	Shoot	*y* = −6.45+$\frac{96.7+6.45}{1+\exp\left(-0.83\left(\log\left(x\right)-\log\left(6.2\right)\right)\right)}$	0.99	0.85 (±0.16)	7.77 (±3.1)	151.2 (±14.6)
	Root	*y* = 7.5+$\frac{83.2-7.5}{1+\exp\left(-2.2\left(\log\left(x\right)-\log\left(4.1\right)\right)\right)}$	0.99	0.89 (±0.11)	4.56 (±0.21)	–
Barnyard grass	Shoot	*y* = 3.3+$\frac{94.7-3.3}{1+\exp\left(-2.2\left(\log\left(x\right)-\log\left(2.7\right)\right)\right)}$	0.99	0.87 (±0.6)	2.77 (0.46)	10.04 (±8.05)
	Root	*y* = 2.95+$\frac{96.3-2.95}{1+\exp\left(-2.1\left(\log\left(x\right)-\log\left(2.4\right)\right)\right)}$	0.99	0.73 (±0.42)	2.43 (±0.28)	8.42 (±4.5)
Red sprangletop	Shoot	*y* = −2.7+$\frac{93.9+2.7}{1+\exp\left(-1.07\left(\log\left(x\right)-\log\left(2.2\right)\right)\right)}$	0.99	0.39 (±0.09)	2.64 (±0.32)	42.7 (±8.7)
	Root	*y* = −5.3+$\frac{90.3+5.3}{1+\exp\left(-1.4\left(\log\left(x\right)-\log\left(1.35\right)\right)\right)}$	0.99	0.42 (±0.17)	1.69 (±0.35)	77.9 (±8.0)

^a^ Using the best‐fit equation based on the *F*‐test, the ED_10_, ED_50_ and ED_90_ values (Concentrations in μM of each allelochemical required for 10, 50, and 90% inhibition of shoot and root growth of tested seedlings) were determined. Abbreviation: SE, standard error. Absence of some ED_10_ and ED_90_ values indicates that these values could not be derived based on the four‐parameter log‐logistic model.

Cress (*Lepidium sativum*) exhibited high sensitivity to NTCT. The ED_50_ values were very low: 0.37 μM for shoots and 0.54 μM for roots. The ED_10_ values were 0.036 μM for shoots and 0.098 μM for roots, indicating that even minimal NTCT exposure leads to significant growth inhibition. The ED_90_ values were 10.8 μM for shoots and 6.38 μM for roots, reflecting a steep dose–response curve where most inhibition occurs over a narrow concentration range.

Lettuce (*Lactuca sativa*) showed considerable sensitivity with ED_50_ values of 1.12 μM for shoots and 0.73 μM for roots. The ED_10_ values were 0.11 μM for shoots and 0.092 μM for roots. The ED_90_ values were 7.75 μM for shoots and 6.56 μM for roots, indicating that while lettuce requires higher NTCT doses for significant inhibition, root growth is somewhat more sensitive than shoot growth.

Amaranth (*Amaranthus retroflexus*) was noticeably less sensitive to NTCT than cress and lettuce, with ED_50_ values of 6.15 μM for shoots and 3.73 μM for roots. However, the ED_10_ value for shoots was 0.003 μM, suggesting some initial sensitivity, but the ED_90_ values were high: 21.10 μM for shoots and 14.5 μM for roots. Thus, substantial NTCT concentrations are necessary to achieve near‐complete inhibition, posing challenges for NTCT‐based weed management.

Timothy (*Phleum pratense*) exhibited moderate sensitivity with ED_50_ values of 2.33 μM for shoots and 3.81 μM for roots. The ED_10_ values were 0.22 μM for shoots and 0.77 μM for roots. The ED_90_ values were 14.7 μM for shoots and 13.9 μM for roots, suggesting that NTCT affects timothy gradually, potentially requiring more frequent or higher doses for effective control.

The two weedy rice accessions (*Oryza sativa* f. *spontanea*) varied in sensitivity. For PI 653426, the ED_50_ values were 10.33 μM for shoots and 5.44 μM for roots, with ED_10_ values of 0.5 μM for shoots and 1.85 μM for roots. For PI 653431, the ED_50_ values were 7.77 μM for shoots and 4.56 μM for roots, with ED_10_ values of 0.85 μM for shoots and 0.89 μM for roots. These results suggest that while NTCT inhibits weedy rice, particularly root growth, higher concentrations or combination treatments may be necessary for effective control.

Barnyardgrass (*Echinochloa crus‐galli*) demonstrated moderate sensitivity, with ED_50_ values of 2.77 μM for shoots and 2.43 μM for roots. The ED_10_ values were 0.87 μM for shoots and 0.73 μM for roots. The ED_90_ values were 10.04 μM for shoots and 8.42 μM for roots, indicating that NTCT can effectively inhibit barnyardgrass at moderate concentrations, but complete suppression may require higher doses.

Red sprangletop (*Leptochloa chinensis*) exhibited ED_50_ values of 2.64 μM for shoots and 1.69 μM for roots. The ED_10_ values were 0.39 μM for shoots and 0.42 μM for roots. The ED_90_ values were notably high: 42.7 μM for shoots and 77.9 μM for roots. These results indicate a gradual inhibitory effect of NTCT, necessitating substantial doses for near‐complete growth suppression. This suggests that effective control of red sprangletop may require higher concentrations of NTCT.

The ED_50_ values represent the concentration at which 50% inhibition of growth is predicted to occur and are critical indicators of the sensitivity of each species to NTCT treatment. They provide a midpoint measure, offering insights into the dosage required to achieve significant inhibition while allowing for comparisons across different species. Based on the ED_50_ values for shoots, the sensitivity of the different entries to NTCT decreased from cress (0.37 μM) to lettuce (1.12 μM), timothy (2.33 μM), red sprangletop (2.64 μM), barnyardgrass (2.77 μM), canola (5.29 μM), amaranth (6.15 μM), weedy rice PI 653431 (7.77 μM), and weedy rice PI 653426 (10.33 μM). The sensitivity based on ED_50_ values for roots was similar with cress (0.54 μM) again being the most sensitive, followed by lettuce (0.73 μM), red sprangletop (1.69 μM), canola (2.40 μM), barnyardgrass (2.43 μM), amaranth (3.73 μM), timothy (3.81 μM), weedy rice PI 653431 (4.56 μM), and weedy rice PI 653426 (5.44 μM).

## 4 DISCUSSION

The use of allelopathy to control weeds may be through natural allelopathic interactions of plants growing in the field or through application of allelochemicals as a natural herbicide. In the former case, some plants with allelopathic potential could be used as cover, mulch and green manure crops to manage weeds through management practices. Allelopathic species may be appropriately rotated or alternated with major crops to manage targeted weeds. Even humus residues can provide desired benefits (Ain et al., 2023; Duke et al., 2000; Einhellig, 1996; Kostina‐Bednarz et al., 2023).

The discovery of the production of NTCT by rice plants, initially identified as a phenylethylamine involved in the defense mechanisms against barnyardgrass and red sprangletop, may open the door for its broader use (Thi et al., 2014). Initially reported as an allelochemical in rice, this study unveils potent inhibitory effects of NTCT on a broad spectrum of weed and test plant species, including amaranth, timothy, weedy rice PI 653426 and PI 653431, barnyardgrass, red sprangletop, canola, cress, and lettuce. As depicted in Figures 1, 2, 3, 4, NTCT inhibits growth in a dose‐dependent manner, with higher concentrations causing increased growth inhibition across all examined plant species.

Our study identified considerable variation in sensitivity to NTCT among the examined plant species. The ED_50_ values for shoots and roots of the nine entries ranged from 0.37 to 10.33 μM for shoots, and from 0.54 to 5.44 μM for roots, respectively (Table 1). Although the relative impact of NTCT on root and shoot growth was not the same in all species, based on ED50 values for both roots and shoots, cress and lettuce were the most sensitive while the two weedy rice PIs were the least sensitive to NTCT. Similar studies have shown that allelopathic compounds may exert selective inhibitory effects on specific weed species while sparing others, highlighting the complex nature of allelopathic interactions (Kong et al., 2019; Thi et al., 2014, 2017; Abbas et al., 2021). The percentage inhibition values obtained in this study corroborate trends observed in similar experiments, emphasizing the reliability and reproducibility of NTCT's phytotoxic properties (Ho et al., 2021).

Our results indicate that NTCT is a particularly potent inhibitor of root growth of cress, lettuce, red sprangletop, canola, and barnyardgrass as their ED_50_ were near or below 2.4 μM. Although not quite as sensitive, ED_50_ for root growth of amaranth, timothy, and the two weedy rice entries were still ≤5.44 μM. NTCT has similar or much greater growth inhibitory effects compared to two allelochemicals isolated previously from Bangladesh rice (*O*. *sativa* L. cv. Kartikshail) by Kato‐Noguchi et al. (2014). The two inhibitors identified by Kato‐Noguchi et al. (2014), 3‐hydroxy‐*β*‐ionone and 9‐hydroxy‐4‐megastigmen‐3‐one, had ED_50_ for root and shoot growth of cress (*Lepidium sativum*) of 4.9 and 9.5 μM, and of 0.54, and 0.72 μM, respectively. In comparison, the ED_50_ we identified for NTCT based on the root and shoot length of cress were 0.54 and 0.37 μM—about 9 and 26 times lower than the ED_50_ of 3‐hydroxy‐*β*‐ionone and equal to and 1.9 times lower than the ED_50_ of 9‐hydroxy‐4‐megastigmen‐3‐one for the root and shoot length of cress.

As described above, the results of the bioassays conducted in this study revealed significant inhibition of root elongation and adverse effects on shoot growth when exposed to NTCT. The varying degrees of root growth inhibition by the different concentrations of NTCT were associated with symptoms such as curled, swollen, or rotting roots and ultimately were associated with plant death. The observed symptoms of root damage, including swelling and rotting, also are consistent with those reported in previous studies investigating the phytotoxic effects of allelochemicals (Ghizlane, 2023). Similar symptoms, including root swelling and necrosis, have been reported in other studies investigating the phytotoxic effects of herbicides and other chemical compounds on plant roots (Duke et al., 2020). The observed root phenotypes suggest that NTCT interferes with essential cellular processes in the root tissues. Previous studies have suggested that NTCT may inhibit root elongation by disrupting microtubule assembly, thereby affecting cell division and cell wall synthesis (Thi et al., 2017). Microtubules are essential for the proper organization of the cytoskeleton and play a crucial role in cell division, elongation, and growth in plant roots (Morejohn et al., 1987). The disruption of cell division and cell wall synthesis in root tissues can lead to abnormal root growth and, ultimately, plant death. However, additional studies are needed to determine the specific mechanisms by which NTCT causes root growth inhibition and associated symptoms.

Based on the described bioassays, NTCT may hold promise for the management of a broad range of weeds, including weeds belonging to the Poaceae family (barnyardgrass, red sprangletop, timothy, and weedy rice) as well as for broadleaf species (amaranth, canola, cress, and lettuce). However, it is important to note that field experiments are needed to explore whether and to what extent NTCT may be able to complement currently used herbicides for the management of weeds in a safe and effective manner. In agricultural fields, root exudation influences chemical and physical properties of soil, microbial community, and growth of competing plants (Li et al., 2019). Our understanding about the diversity of compounds and the mechanisms involved in exudation/release of compounds from root cells of plants is limited. However, research indicates that plants can release a large range of compounds through plasmalemma or endoplasmic‐derived exudation and proton‐pumping mechanisms (Abbas et al. 2021). Indeed, rice can produce and release different allelochemicals that have various biological effects into its surroundings, and allelopathic behavior has been shown to be influenced by rice variety and origin (Dilday et al., 2001; Khanh et al., 2007; Kato‐Noguchi, Salam & Suenaga, 2011). Bioassay results suggest that NTCT, which was originally identified in OM 5930 rice plants, may have allelopathic effects if released into the soil in sufficiently high concentrations. However, a better understanding of NTCT release, interactions, and persistence in the soils of rice fields is vital in order to evaluate its utility as an allelopathic compound and to design strategies to enhance weed suppression and increase rice yields in a sustainable manner.

Beyond the immediate challenges of yield reduction, the economic consequences of infestations of weedy rice such as PI 653426 and PI 653431 are noteworthy. Farmers face escalating costs associated with the need for more intensive weed control measures, including additional herbicides and labor. Increasing economic burdens require a paradigm shift towards sustainable and integrated weed management strategies (Burgos et al., 2008, 2014). This is important not only for individual farmers but also for the overall economic viability of rice farming in many countries. In addition to negative impacts on yield through competition, the presence of weedy rice also influences the marketability of harvested rice crops as rice contamination with weedy rice grains compromises the quality of the product, impacting market value and consumer acceptability. With increasing pressure from weedy rice, meeting stringent quality standards for rice products becomes a challenge for farmers, emphasizing the urgency for effective weedy rice management strategies to safeguard both economic interests and market competitiveness (Jia & Gealy, 2018). While the sensitivity of weedy rice to NTCT is considerably lower than that of other species, NTCT nonetheless dramatically reduced both shoot and root growth of weedy rice at concentrations ranging between 4.56 and 10.33 μM (ED_50_) in our bioassays. Continued research is needed to establish whether NTCT can be utilized to combat weedy rice in rice fields.

In summary, the results of this study provide important insights into the growth inhibotory effects of NTCT on shoot and root growth of diverse plant species. NTCT demonstrated a dose‐dependent inhibitory effect on both shoot and root growth across a variety of species, including barnyardgrass, red sprangletop, timothy, weedy rice, amaranth, canola, cress, and lettuce. The observed symptoms, including root damage and plant death, suggest that NTCT may disrupt essential cellular processes within root tissues. These findings highlight NTCT's potential efficacy in suppressing the growth of a diverse range of weeds, including both grasses and broadleaf species. While these results indicate that NTCT could contribute to sustainable weed management, particularly in controlling herbicide‐resistant weeds in various ecological settings, further research is essential. Future studies should focus on fully elucidating the mechanisms by which NTCT inhibits growth, its efficacy under field conditions, and on understanding potential broader implications for sustainable weed management.

## CONFLICT OF INTEREST STATEMENT

No potential conflict of interest was reported by the author(s).

## Supporting information


**Data S1:** Supporting Information.

## Data Availability

The datasets generated and analyzed during the current study are available in the Figshare repository: 10.6084/m9.figshare.26964919.
